# Electroacupuncture for Decorticate Rigidity of the Upper Limbs in a Patient with Anoxic Brain Damage

**DOI:** 10.1155/2013/524603

**Published:** 2013-10-01

**Authors:** WooSang Jung, SeungWon Kwon, SeongUk Park, SangKwan Moon, JungMi Park, ChangNam Ko, KiHo Cho

**Affiliations:** ^1^Department of Cardiovascular and Neurological Diseases, Kyung Hee University Oriental Medicine Hospital, 1 Hoegi-dong, Dongdaemun-gu, Seoul 130-702, Republic of Korea; ^2^Department of Cardiovascular and Neurologic Diseases, Kyung Hee University Hospital at Gangdong, 149, Sangil-dong, Seoul, Republic of Korea

## Abstract

Decorticate rigidity is a type of abnormal posturing that can make it difficult to move a patient and to change the patient's position to prevent a decubitus ulcer. This condition poses a latent risk of bed sores. To prevent those complications, we used electroacupuncutre for decorticate rigidity of the upper limbs in a patient with anoxic brain damage. A 51-year-old man complained of rigidity of both of the upper and lower extremities due to anoxic brain damage. His rigidity was exhibited as flexed arms and extended legs, which are the typical positions in decorticate rigidity. Prior to electroacupuncture, his decorticate rigidity was treated with dantrolene sodium and baclofen. However, his rigidity had not improved. This patient received total 41 sessions of electroacupuncture. The patient's Modified Ashworth's Scale changed from 4 at baseline to 2 after the treatment, indicating an improvement in the rigidity of the upper limbs. Preston's Hypertonicity Scale and passive ROM of the elbow joints also improved. We report the observed effects of electroacupuncture for decorticate rigidity of the upper limbs in a patient with anoxic brain damage. Further controlled studies are needed to determine whether electroacupuncture is a useful alternative treatment for decorticate rigidity in patients with anoxic brain damage.

## 1. Introduction

We report a case of decorticated rigidity due to anoxic brain damage that was successfully treated with electro-acupuncture combined with conventional therapeutic options such as muscle relaxants and physical manual therapy.

## 2. Case History

### 2.1. Patient Characteristics and Diagnosis

**Figure 1:**
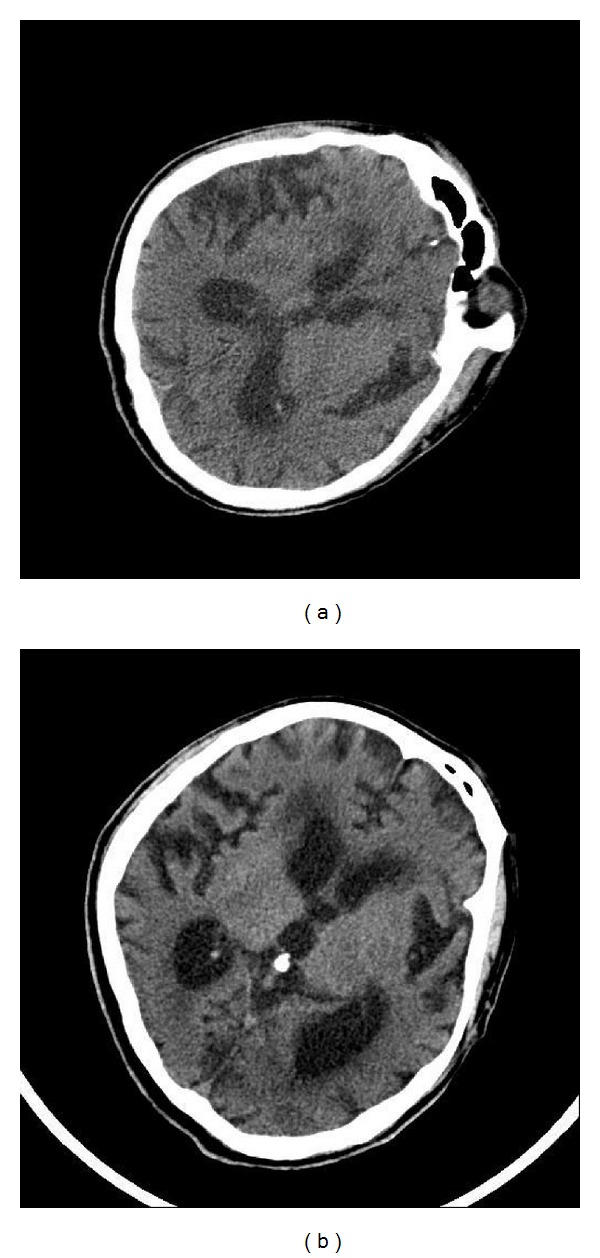
The brain computed tomography (CT) image at onset and admission time. (a) Brain CT image at onset and (b) brain CT image at admission time (3 months after onset).

 A 51-year-old man complaining of rigidity of both of the upper and lower extremities due to anoxic brain damage was admitted to the Department of Cardiovascular and Neurological Diseases of Kyung Hee University Oriental Medicine Hospital. This patient had collapsed during a marathon and had received cardiopulmonary resuscitation (CPR). The doctors recognized acute myocardial infarction (MI) and provided conventional therapies for this condition. However, anoxic brain damage was caused by insufficient blood supply to the brain. He manifested rigidity, dyspnea, and dysphagia. His rigidity was exhibited as flexed arms and extended legs, which are the typical positions in decorticate rigidity. Although this patient had underlying hypertension and diabetes mellitus, he and his family had not recognized these conditions until onset of MI. At admission, he revealed GCS score 7 (E4 V0 M3) and used tracheostomy tube, nasogastric tube. The brain computed tomography (CT) image taken at admission (Figure 1) revealed severe cerebral cortical atrophy. Laboratory data did not reveal abnormal values for all items. He was admitted to Kyung Hee University Oriental Medicine Hospital 3 months after onset. Prior to his admission, his decorticate rigidity was treated with dantrolene sodium (25 mg) and baclofen (5 mg). However, his rigidity had not improved, and his condition had remained the same since onset.

### 2.2. Treatment

Electro-acupuncture was performed as a therapy for symptoms of decorticated rigidity of the upper limbs. Daily electro-acupuncture treatment was performed between 14:00 and 15:00 for 41 consecutive days (PG-306, Japan). The acupoints used in this case were LI11, LI10, TE5, and LI4, bilaterally. The following conditions were used: stimulation wave, biphasic (60 Hz); pulse duration, 0.4 ms; and stimulation strength, low. The electrical stimulation power was the same for each treatment (Pulse Generator PG-306, Suzuki Inc., Japan). The patient's elbow joints and wrists showed mild movement during electro-acupuncture therapy. Each treatment procedure lasted 20 min and was performed by skilled specialists (Korean Medicine Doctor (KMD)) by using stainless steel acupuncture needles (Dongbang Acupuncture Inc., Korea; 40 mm (length) × 0.25 mm (diameter)). Furthermore, Bojungikgitang, a Korean herbal medicine decoction, is administered to control general condition of this patient. There was no change in total daily dose of dantrolene sodium and baclofen before and after electro-acupuncture treatment.

### 2.3. Evaluation Methods

 We used 3 methods to evaluate the effect of electro-acupuncture on the patient's rigidity: the Modified Ashworth's Scale (MAS) (Table 1) [1], Preston's Hypertonicity Scale (Table 2) [2], and the measurement of the passive range of motion (ROM) of the elbow joints. 

### 2.4. Course of Symptoms

**Figure 2:**
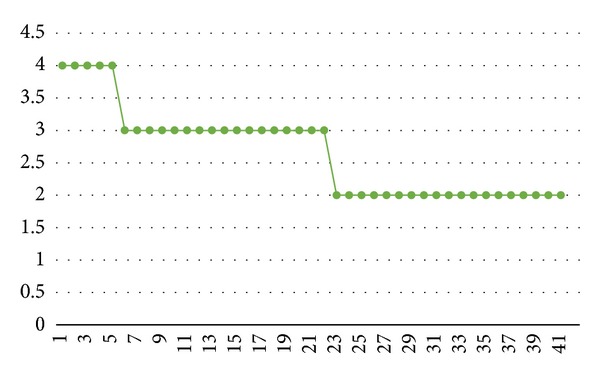
MAS score changes. MAS: Modified Ashworth's Scale; *X*: numbers of sessions; *Y*: MAS score.

**Figure 3:**
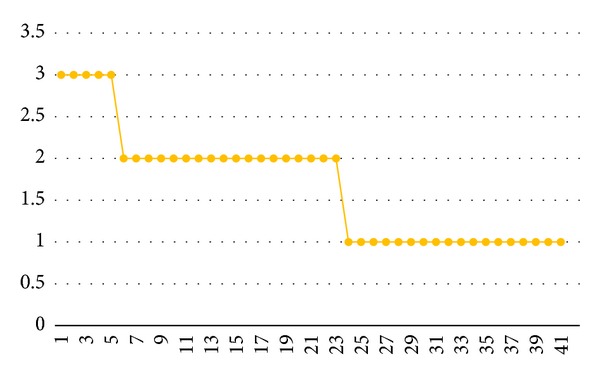
Preston's Hypertonicity Scale changes. *X*: numbers of sessions; *Y*: Preston's Hypertonicity Scale score.

The patient was treated for 41 days, and the results are presented graphically in Figures 1, 2, and 3. The initial MAS score of 4 improved slightly during the procedure: the first improvement in this score (3 points) was observed after the 6th treatment, and a further improvement to 2 points was noted by the time of the 24th treatment. The MAS score remained at 2 points at the last session (Figure 2). The initial Preston's Hypertonicity Scale score of 3 fell to 2 points after the 6th treatment. Preston's Hypertonicity Scale score of 1 was noted at the time of the 24th treatment, and this value remained unchanged until the last treatment (Figure 3).

**Figure 4:**
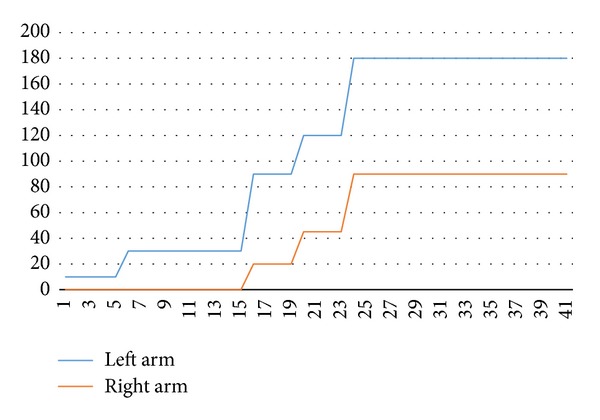
Passive ROM changes of both elbow joints. ROM: range of motion; *X*: numbers of sessions; *Y*: passive ROM of both elbows.

Before treatment, the passive ROM of the left elbow joint was 10°, and that of right elbow joint was 0°. With continuous electro-acupuncture therapy, the passive ROM of both elbow joints improved. Approximately 24 days after initiating treatment, the passive ROM of left elbow joint increased to 180° and the passive ROM of the right elbow joint increased to 90°. The passive ROMs of the left elbow joint and the right elbow joint remained constant at 180° and 90°, respectively, until the last treatment (Figure 4).

## 3. Discussion

 Decorticate rigidity is a type of abnormal posturing. Abnormal posturing is an involuntary flexion or extension of the arms and legs, indicating severe brain injury. Decorticate rigidity exhibited as flexion of the upper limbs and extension of the lower limbs indicates damage in the cerebral hemispheres, the internal capsule, and the thalamus. Our patient's rigidity was a result of anoxic brain damage due to myocardial infarction. 

 This patient exhibited flexed arms and extended legs, which are the typical positions in decorticated rigidity. This patient was diagnosed with anoxic brain damage on the basis of his medical history, symptoms, and brain imaging. Although he had been treated with conventional therapies for rigidity (muscle relaxants and physical therapy) after diagnosis, his symptoms did not improve before he was admitted to KyungHee University Oriental Medicine Hospital. The patient's family and caregiver had trouble in moving the patient owing to his severe rigidity. Furthermore, his rigidity made it difficult to change his position for preventing the formation of decubitus ulcers, and this patient was at latent risk of bedsores. 

Several studies have shown that electro-acupuncture can treat rigidity [3, 4]. One of these studies [4] has suggested that high-frequency and low-strength electro-acupuncture on LI11, LI10, TE5, and LI4 can reduce rigidity of the upper limbs in stroke patients. Because the rigidity of the upper limbs of stroke patients exhibits a flexion, the authors of this study used acupoints LI11, LI10, TE5, and LI4 to stimulate the arm extensor muscle group, which is an antagonist of the arm flexor muscle group. Although the diagnosis of the present case was anoxic brain damage, we observed a form of rigidity in this patient that was similar to that in stroke patients, namely, flexion of the upper limbs. As mentioned above, conventional therapy did not improve his rigidity. Thus, we used electro-acupuncture therapy based on the previous study in stroke patients (acupoints LI11, LI10, TE5, and LI4 and a high-frequency/low strength stimulation method (60 Hz, 0.4 ms pulse duration, and low strength)) to relieve our patient's arm rigidity symptoms.

 In the present study, the patient's upper limb rigidity gradually improved during the follow-up period. The MAS score and Preston's Hypertonicity Scale improved at both the 6th and 24th sessions. The passive ROM of both elbow joints also improved during the 41 sessions, and notably, the passive ROM of the left elbow joint showed a normal value at the endpoint. Therefore, we believe that electro-acupuncture was effective for treating this patient's upper limb rigidity.

 In general, anoxic brain damage which results in coma causes a vegetative state, and these patients will remain in a vegetative state until death. Therefore, prognosis of anoxic brain damage is very poor. And patients who reveal decorticate rigidity are in a coma or vegetative state and have poor prognoses, with risks for cardiac or respiratory arrest [5]. However, in this case, there were improvements in upper limbs rigidity due to anoxic brain damage. Thus, we could make it easy to move a patient and to change the patient's position to prevent a decubitus ulcer and related complications such as pneumonia. We think that the use of electro-acupuncture for decorticate rigidity not only decreased tone of muscle of upper limbs but also prevented complications.

Baclofen is used to treat rigidity in conventional drug treatments [6, 7]. However, patients who have nephropathy cannot use baclofen owing to its toxicity; moreover, a previous study [8] has suggested that patients on dialysis are exposed to nephrotoxicity during baclofen intake. Therefore, baclofen cannot be used to treat rigidity in patients who have nephropathy. We believe that electro-acupuncture may be an alternative for treating decorticate rigidity in elderly people undergoing dialysis. In our case, treatment with conventional therapy (drugs and physical therapy) for decorticate rigidity did not result in improvement, but improvement was noted in the rigidity of the upper limbs after treatment with electro-acupuncture. Therefore, we propose that electro-acupuncture is a therapeutic option in patients who show no improvement despite therapy.

A limitation of the present case study is that we could not treat the rigidity of the lower limbs. The patient's lower limbs exhibited extension, but we could not find any studies in the literature that provided therapeutic methods for lower-limb rigidity. In addition, because the symptoms of the lower limbs were less severe than those of the upper limbs at the time of admission, we concentrated our efforts on treating the rigidity of the upper limbs. Studies that provide data about treating lower-limb rigidity are required in the future. Furthermore, additional research is needed to determine the efficacy of electro-acupuncture on decorticate rigidity.

 In summary, 41 days of electro-acupuncture treatment of LI11, LI10, TE5, and LI4 improved the rigidity of the upper limbs in patients with anoxic brain damage. Although it is difficult to draw conclusions based on a case study of only 1 patient, we propose that further studies would help determine whether electro-acupuncture is a plausible alternative in the treatment of decorticate rigidity of the upper limbs in patients with anoxic brain damage.

## Figures and Tables

**Table 1:** The Modified Ashworth's Scale for grading spasticity [1].

Grade	Definition
G0	No increase in muscle tone.
G1	Slight increase in muscle tone, manifested by a catch and release or by minimal resistance at the end of the range of motion when the affected part(s) is moved in flexion or extension.
G1+	Slight increase in muscle tone, manifested by a catch, followed by minimal resistance throughout the reminder (less than half) of the ROM.
G2	More marked increase in muscle tone through most of the ROM, but affected part(s) easily moved.
G3	Considerable increase in muscle tone passive movement difficult.
G4	Affected part(s) rigid in flexion or extension.

**Table 2:** Preston's Hypertonicity Scale [2].

Grade	Definition
G0	No abnormal tone detected during slow, passive movement.
G1	Mild: first tone or resistance is felt when the muscle is in a lengthened position during slow passive movement.
G2	Moderate: first tone or resistance is felt in the midrange of the muscle during slow passive movement.
G3	Severe: first tone or resistance is felt when the muscle is in a shortened range during slow passive movement.
